# Rigid and Flexible Double Shear Lap Adhesive Joint at Elevated Temperature—An Experimental Study

**DOI:** 10.3390/polym13172873

**Published:** 2021-08-27

**Authors:** Klaudia Śliwa-Wieczorek, Bogusław Zając

**Affiliations:** Faculty of Civil Engineering, Cracow University of Technology, 24 Warszawska Str., 31-155 Cracow, Poland; bozajac@pk.edu.pl

**Keywords:** elevated temperature, double shear lap joint, glass transition, epoxy, polyurethanes, rigid and flexible

## Abstract

Double lap adhesive connections made of Sika^®^ PS and Monolith EP2579-1 were studied experimentally in shear tests. The destructive shear tests were conducted under a quasi-static load at 20 °C and 80 °C. The aim was to study the impact of elevated temperature on the load capacity of the joint and make a comparative analysis of the results for two types of adhesives: polyurethane Sika^®^ PS (flexible) and epoxy Monolit EP 2579-1 (rigid). The impact of adhesive layer thickness (t = 1, 2 and 4 mm) on the structural response of the joint was tested in two temperature ranges. A distinct impact of the temperature on the joint deformability was noticed. A visual assessment of the joint failure was performed and the initiation and form of failure was described. At 20 °C, the ultimate loading for epoxy adhesive joint depending on the joint thickness (t) was greater than for the polyurethane joint by, respectively, 282% for t = 1 mm, 88% for t = 2 mm and 279% for t = 4 mm. It was proved that the temperature increases to 80 °C in case of both adhesives reduces the mean destructive force in comparison with the measurements made at 20 °C. For the Sika^®^ PS (PUR two-component polyurethane) adhesive, the greatest load capacity decrease was measured for the joint of thickness t = 2 mm (55%), and in case of the epoxy adhesive for the joint of thickness t = 4 mm (89%). It was found that after reaching the destructive force the flexible joints retain a partial load capacity contrary to the rigid joints.

## 1. Introduction

Adhesives are important for industries that shape the everyday life of people. They are widely used, mainly in the automotive and aerospace industries, and find application also in medicine, electronics and civil engineering. Their huge potential makes the researchers look for more effective improvements of already know solutions and for innovative solutions. The main advantages of adhesive bonding include an even distribution of stresses (the load can be distributed over a larger contact area than in a pin-like fastener connection where the load transfer takes place in the embedment zone of fasteners where radial oriented embedment stresses are emerging and perpendicular oriented stress components is loading the timber members in tensile stresses perpendicular that may initiate splitting-failure of timber connection), resistance to corrosion, possibility of joining components of various shapes, absence of weakening of the joined components by drilling holes in them and good strength-to-weight ratio [1,2,3].

The studies are also conducted on the improvement of mechanical adhesive and rheological properties as well as improvement of durability and resistance to damage at fatigue loads of adhesives and adhesive joints by using various reinforcements and additives in the form of micro- and nanoparticles [4,5,6,7]. With environmental aspects in mind, attempts are made to improve the properties of adhesives by using additives of natural origin in the form of micro- and nanofibers deposited in the adhesive joint [8,9,10]. Delzendehrooy et al. [10] proved that the strength of lap joints reinforced with Rachis fibres in the amount of 2% by weight, which were previously treated with a NaOH solution, increased by 140%. There are also attempts to develop new adhesives formulas based on natural materials such as linseed oil [11].

In construction, adhesives are widely used in manufacture of wooden bar and board parts and wood-based materials [12,13,14,15]. However, as a result of being able to join different and complex materials with various substrate types [16], the adhesives are also used in modern methods of repair, reinforcement and protection of structures. The reinforcement technology usually involves bonding of, steel bars, sheets (flat bars) or fibre-reinforced polymer composites in the tension zone of a component using epoxy adhesives [17,18,19,20]. Flexible adhesives on the other hand find application mostly in repair of masonry and concrete structures [3,21] and seismic protection of buildings where dissipation of energy and reduction of edge stresses are desirable [22,23]. Lasowicz et al. [24] studied the effectiveness of flexible polyurethane adhesive in vibration reduction of a structure subjected to dynamic loads. The authors proved that the use of polymer adhesive is an effective method of vibration reduction in the studied frequency range up to 40 Hz. Ref. [25] deals with the durability of the PS polyurethane adhesive joint used for the structure reinforcement.

**Figure 1:**
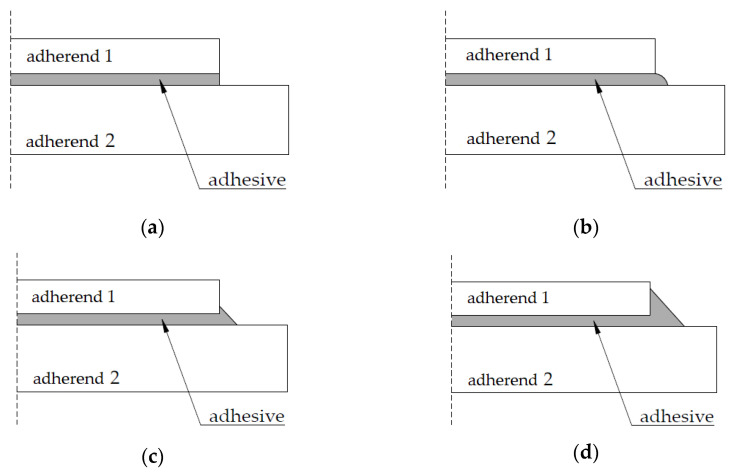
Geometric parameters of adhesive layers: (**a**) square edge; (**b**) rounded spew fillet; (**c**) small spew fillet; (**d**) full spew fillet.

Literature sources have identified many factors affecting the durability of adhesive joints, which generally can be divided into three main categories: materials, stresses and environment. The materials category describes the impact of inclusions and discontinuity which can take place during the construction of the joint on the subsequent distributions of stresses [26]. The impact of the joint finish (square edge, rounded spew fillet, small spew fillet, full spew fillet, as shown in Figure 1 below) on its load capacity was studied [27,28].

A few researchers dealt with the impact of adhesive layer geometry and surface preparation before bonding on the structural response of the joints [29,30,31,32]. Vallée et al. [29] conducted experimental and analytical studies of the double lap joint comprising GFRP (Glass Fibre Reinforced Polymer) profiles and polyurethane adhesive. The aim was to find the optimum joint thickness (i.e., the thickness for which the mean breaking stresses are the highest). The range of studied joint thickness was 0.3–10.0 mm and of the joint length 50–200 mm. The authors proved that the optimum adhesive joint thickness was 1.0 mm and that the joint strength increased with the lap length. Liao et al. [33] includes an analysis of the adhesive layer thickness and type and scarf angle on the behaviour of a scarf adhesive joint subjected to uniaxial tensile loading, focusing additionally on the failure energy. It was proved that for brittle adhesives the failure energy increases as the adhesive layer thickness decreases. However, this relationship does not apply to all adhesives. The failure energy understood as the area under the diagram, may increase with the increase of adhesive thickness, because the joint retains its partial load-bearing capacity. For the joint with the brittle adhesive, after reaching the peak, the maximum load F_max_ drops to zero sharply with less deformation. If we increase the joint thickness, we can observe behaviour similar to flexible adhesives, i.e., the added loading decreases quite slowly experiencing long displacement until to final failure. Additionally, in the article, the researchers noted that the effect of the adhesive thickness is more noticeable with decreasing the scarf angle.

In the environment category, the research focused on the impact of humidity and temperature on the properties of adhesives and joints [34,35,36,37,38,39,40]. Moussa et al. [41] subjected epoxy resins to thermal tests. The authors observed the impact of cyclic heating of such adhesives to temperature above the glass transition temperature and then cooling back to the room temperature in the context of possible weakening of the adhesive joint. The conclusions indicated the absence of material degradation, restoration and even improvement of the initial mechanical properties after cooling due to post-curing of the material. A very extensive study on shear adhesive joints, rigid and flexible, operating at elevated temperature were conducted by [42]. The author conducted strength testing and numerical analysis of single shear joints with the use of a carbon fibre strip (S512) and three different substrates: brick, concrete and wood. The specimens were tested at 20, 40, 60 and 80 °C. The behaviour of rigid epoxy joints and of flexible joints was compared, indicating a favourable impact of flexible joints on the reduction of stress concentrations and redistribution of stresses over a larger area.

The estimation of stress distribution in an adhesive layer is a separate research area. The simplest, classical analysis of a single lap joint assumes that the adhesive layer deforms only by shearing, and the shear stress is constant on the joint length and expressed by the formula:

(1) $$\tau =\frac{P}{bl}$$

where: *P*—load on the joint, *b*—adhesive layer width and *l*—adhesive layer length.

New models emerged that accounted for impact of various factors such as: occurrence of the deformation of glued components along the lap length caused by tension (Volkersen model [43]); occurrence, in addition to tensile force P, of the bending moment M and transversal force V applied to the joint ends (Goland and Reissmer model [44]); or the impact of large deformation (Hart-Smith model [45]). The detailed review of analytical models for adhesive joints was presented by da Silva et al. [46,47]. In their analyses, they included such aspects as conformity of the obtained results of numerical analyses with the results obtained in experiments, theory complexity degree, and the type of analysis (simplest linear elastic or nonlinear elasto-plastic). The conclusions indicate that the design process should account for both the geometry and temperature (particularly for epoxy joints which can soften and lose strength at temperatures above the glass transition temperature Tg) at which the joint will operate. This undoubtedly defines the safety level, and because there is no single standard method of designing adhesive joints, more studies are helpful for a better understanding of their behaviour in different conditions during use.

In thispaper, the authors proposed two selected temperatures: 20 °C and 80 °C, which could be the limits of a real temperature range that can occur during use as a result of seasonal changes or exposure of the components to sunlight, e.g., in transparent roof structures.

The tests were conducted with two-component polyurethane flexible adhesive Sika^®^ PS, whose us was described in [48]. The adhesive was used in wood-glass girders subjected to multiple variable loads. The tests were carried out at 20 °C. The author proved that polyurethane adhesives used to join glass with wood have a greater fatigue strength than the stresses caused by operational load. Nevertheless, the studies broaden the knowledge on the use of proposed polyurethane for joining wooden structures. The other adhesive was EP 2579-1. Although it is a rigid epoxy adhesive, it showed properties that can favourably affect the wood-wood adhesive joints which is described further in the paper.

The aim of the paper was to study the impact of elevated temperature on the load capacity of the adhesive joint and make a comparative analysis of the results for two types of adhesives: polyurethane (flexible) and epoxy (rigid). The other aim was to study the impact of adhesive layer thickness (t = 1, 2 and 4 mm) on the structural response of the adhesive connection at two temperatures 20 and 80 °C (±2 °C). The paper presents the directions of further research that would allow an effective choice of the adhesive type and thickness and broaden the current knowledge, increasing the safety of designed adhesive joints.

## 2. Materials and Methods

### 2.1. Tests

**Figure 2:**
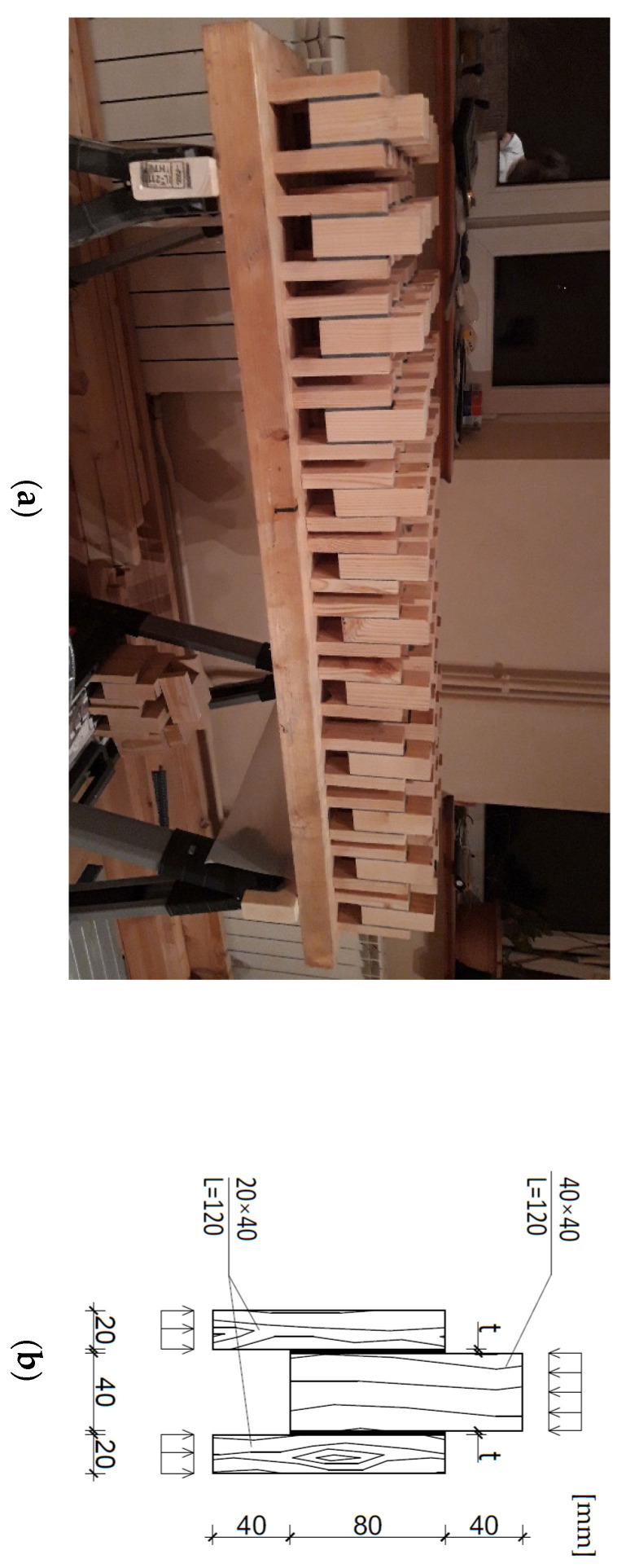
Double lap joint: (**a**) prepared specimens; (**b**) specimen dimensions, where: t—adhesive layer thickness.

The preparation involved making a double lap joint with three adhesive layer thicknesses (t = 1, 2 and 4 mm, respectively), with adhesive bonding line length of 80 mm and width of 40 mm. Six test specimens were made for each thickness; 72 specimens in total were tested in the experiment. The specimens prepared for the test and the specimen dimensions are shown in Figure 2a,b. Two adhesives were used: flexible two-component polyurethane from Sika^®^ of brand name Sika^®^ PS and two-component cold-curing epoxy adhesive Monlith^®^ EP 2579-1. The strength tests of the materials forming the joint: wood and adhesive were performed at two temperatures: 20 °C and 80 °C (±2 °C).

### 2.2. Wood

All specimens were made of the Douglas wood (*Pseudotsuga menziesii (Mirb.) Franco*) for which the manufacturer declared the strength class C24 (C24—strength class for softwood based on tension test) according to PN-EN 338:2016 [49] and the basic characteristic strength parameters were: bending strength f_m,k_ = 24 MPa; tensile strength parallel (0) to grain f_t,0,k_ = 14.5 MPa; tensile strength perpendicular (90) to grain f_t,90, k_ = 0.4 MPa; compression strength parallel to grain f_c,0,k_ = 21 MPa; compression strength perpendicular to grain f_c,90,k_ = 2.5 MPa; shear strength f_v,k_ = 4.0 MPa. The elastic parameters are: mean modulus of elasticity E_0,mean_ = 11 GPa and mean shear modulus G_mean_ = 0.69 GPa). The authors verified the strength parameters in the bending test, tensile test parallel and perpendicular to the wood grain, shear test and compression test parallel to the grain. All tests on the specimens were conducted at two temperatures, 20 °C and 80° C (±2 °C). The wood density was ρ = 490–510 kg/m^3^ with mean moisture content of 8.9% at 20 °C. The paper presents the experimental result for compression test parallel to the grain at 20 °C and 80 °C.

### 2.3. Adhesive

Two adhesives were used in the tests: flexible polyurethane Sika^®^ PS and rigid epoxy EP 2579-1. The Sika^®^ PS is recommended for flexible joining of structural parts and making protective coatings. Currently, its main use is the reinforcement and repair of concrete and masonry structures (the repair method is protected by patent PL207028 (B1)), particularly in seismic areas which has been widely described in [3]. Before testing, all specimens were conditioned for 21 days in a room at stable temperature of 20 °C and humidity of 65% ± 5%.

The following tests were performed for adhesives at 20 °C and 80 °C:

- Uniaxial tensile test of dumbbell-shaped specimens in accordance with ASTM D-638-03: 2004 Standard test method for tensile properties of plastics [50] and PN-EN ISO 527-2:2012 [51] Plastics. Determination of tensile properties. Test conditions for moulding and extrusion plastics. The specimen length was 152 mm, measuring base 50 mm, and thickness 4 mm;
- Uniaxial compression test on cylindrical specimens of diameter 28 mm and height 28 mm. The specimens were subjected to quasi-static load in accordance with the recommendations of ASTM D 695-02a: 2002 Standard test method for compressive properties of rigid plastics [52];
- Adhesive hardness measurement using a manual Zwick Roell hardness tester (on the Shore A scale from 1 to 100).

The purpose of the tests was a comparative analysis of material behaviour at elevated temperature, below and above the glass transition temperature in case of the Monlith^®^ EP 2579-1 epoxy adhesive. The Sika^®^ PS adhesive in the tests was in the rubbery state above its glass transition temperature Tg. The occurring structural changes affect the mechanical parameters, and the relationships should be accounted for in the design process of joints in a wide range of operating temperatures. The technical data of the tested adhesives taken from the manufacturer are presented in Table 1.

### 2.4. Manufacturing

The specimens composed of outer lamellas (solid Douglas wood/*Pseudotsuga menziesii*), class C24 (C24—strength class for softwood based on tension test), cross section 20 mm × 40 mm and length 120 mm and the central lamella: cross section 40 mm × 40 mm and length 120 mm were glued together using three different adhesive layer thicknesses, t = 1 mm, t = 2 mm and t = 4 mm. In order to keep the precise dimensions, a bonding in mould method was developed. The specimens were made in groups of 8–10 pcs in a single mould. The mould allowed obtaining the desired adhesive layer thickness: 1 mm, 2 mm or 4 mm. For the specimens bonded with SIKA^®^ PS, the joined surfaces had to be suitably prepared before the application of the adhesive. The cleaned and dust-free surface was primed using the SIKA^®^ ZP Primer. SIKA^®^ ZP Primer is a solvent-based epoxy resin compound, 1-component primer. Density 1.0 kg/dm3, application temperature +5 °C to 35 °C. Due to its low viscosity, the Sika^®^ PS adhesive was poured into the moulds and it spread gravitationally. According to the datasheet, the EP 2579-1 adhesive does not need the surface priming. After mixing, the epoxy resin was applied using a spatula.

**Figure 3:**
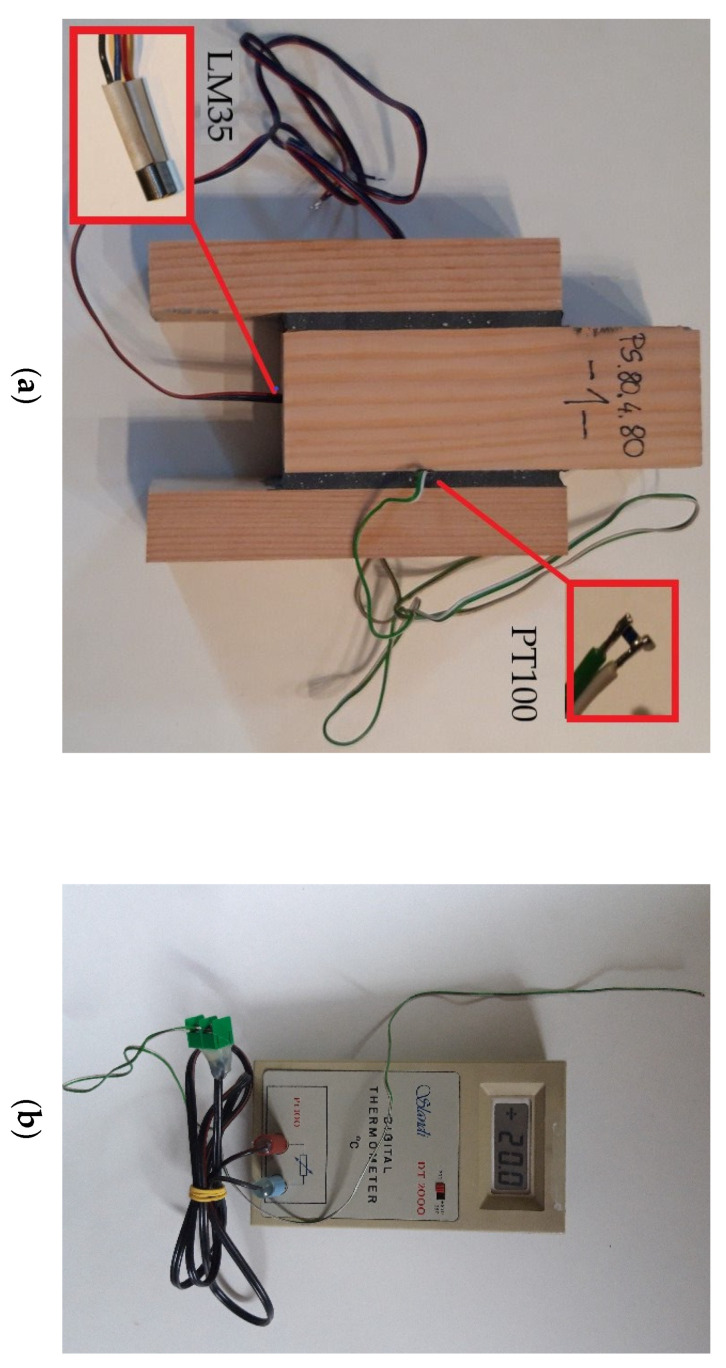
Temperature verification in a double lap joint: (**a**) specimen with sensors prepared for testing at 80 °C; (**b**) measuring instrument for temperature control in the adhesive joint.

Two electronic sensors were used to measure the temperature. In selected specimens, a PT 100 thermocouple (manufacturer VISHAY Electronic GmbH, Germany) was placed inside the adhesive joint. Due to its small dimensions of 0.45 mm × 0.85 mm × 1.55 mm, this sensor did not disturb the obtained results (very low heat capacity). The temperature inside the wood was measured using LM35 sensors (manufacturer Texas Instruments) placed in the central lamella, in a hole with a diameter of 5.5 mm. The specimen with sensors and the measuring instrument is shown in Figure 3a,b. After application of the adhesive on the wood surface, all moulds were pressed using 8–10 bolt clamps. The pressure was kept for minimum 24 h. Before testing, the specimens were seasoned for 21 days in a room at stable temperature of 20 °C and humidity of 65% (±5%).

### 2.5. Testing Procedure

**Figure 4:**
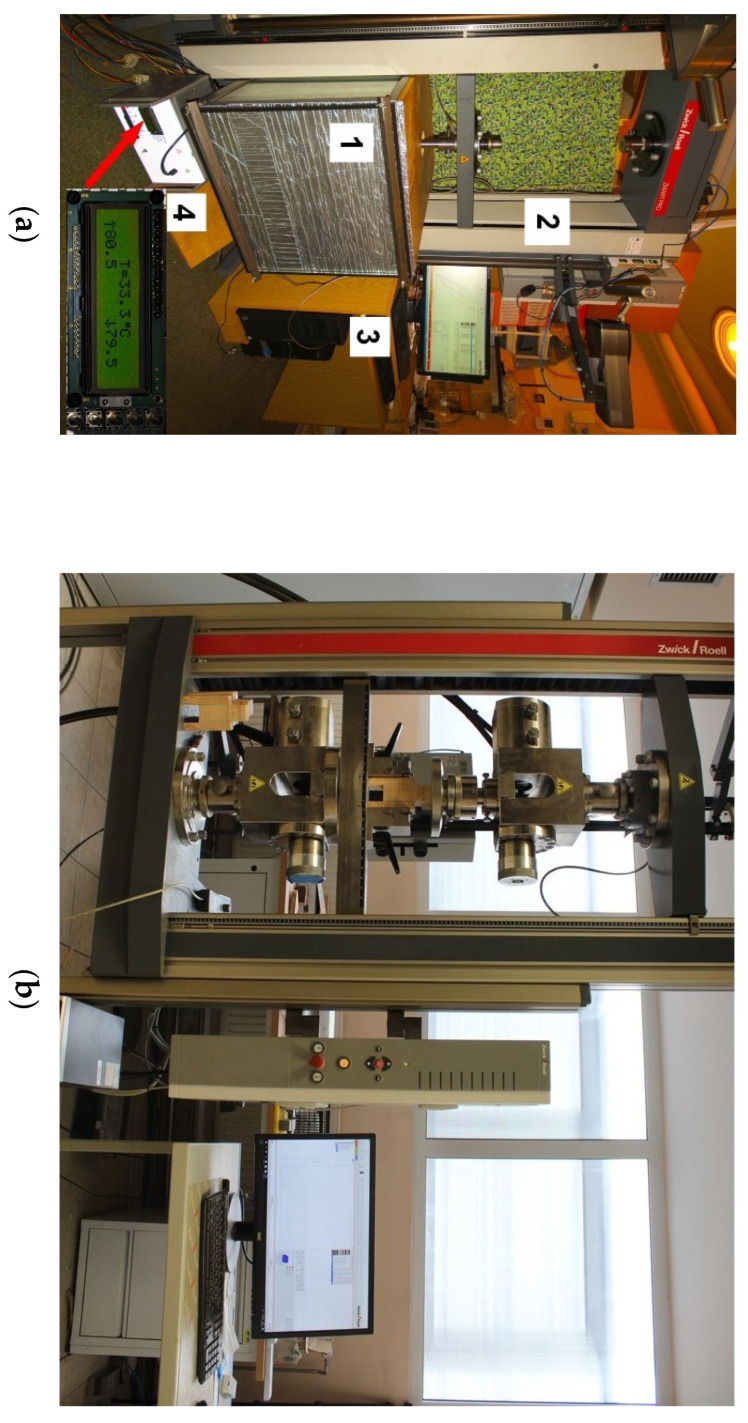
Test configuration details: (**a**) Zwick 1455 test stand: 1—temperature chamber, 2—strength tester, 3—control computer, 4—temperature regulator with power supply; (**b**) Zwick 100 test stand.

The experiment was conducted on specimens compressed as shown in Figure 4b. The load was increased by a constant movement of the cross-beam whose speed, depending on the test, was: 1 mm/min, 2 mm/min and 4 mm/min. The test was conducted until the specimen was destroyed. Lateral barriers protecting against displacement shown in Figure 4b were used to limit the effect of buckling. The test was conducted on a Zwick 1455 tester with the maximum force of 20 kN and on a Zwick 100 tester for specimens glued with the epoxy adhesive and tested at 20 °C (because the range of the Zwick 1455 was exceeded).

The tests at 80 °C were conducted in a temperature chamber with power supply adjustable up to 1000 W, which allows the temperature up to 100 °C to be reached. The temperature was stabilized by means of a digital regulator with a 0.5 °C resolution and electronic power changeover switches. The thermal chamber is presented in Figure 4a. The specimens were heated and stabilized in the chamber for 6–8 h until the set temperature was reached and stabilized. The temperature was measured with the 0.1 °C accuracy inside the wood and inside the adhesive layer. The displacement of the machine cross-beam and the force increase were measured during the test. The form of specimen failure and impact of the adhesive layer thickness of the joint load capacity were analysed.

## 3. Results and Discussion

The test results and the comparative analysis are presented below. The results are presented for material testing (tests of joint components) and for the double shear lap joints in the compression test. The impact of the test speed and temperature on the behaviour of the adhesive, and the impact of the temperature on decrease of the wood compressive strength parallel to grain were described. Impact of the adhesive layer thickness and temperature on the joint load capacity and form of failure was presented for double shear lap joints.

### 3.1. Material Testing (Adhesives and Wood)

The static tensile test on dumbbell-shaped specimens included six measurements for each speed (72 specimens in total). The specimen length was 152 mm, measuring base 50 mm, and thickness 4 mm. Before testing, all specimens were conditioned for 21 days in a room at stable temperature of 20 °C and humidity of 65% ± 5%. The adhesive was mixed as described in a product data sheet. The mixing ratio for Sika^®^ PS A:B = 100:11 parts by weight. The adhesive was mixed for 60 to 80 s, the speed of mechanical mixing was 600–800 rpm. The mixing ratio for epoxy EP 2579-1 component A:B = 1:1 (by weight). Mean values (m) of tensile stress and deformation, standard deviation (s) and coefficient of variation (V) were calculated for each series and are presented in Table 2. The impact of deformation and temperature on the material behaviour was studied. The results presented in Table 2 include information on three deformation speeds: 10^−1^ mm/min, 10^0^ mm/min and 10^1^ mm/min at 20 °C and 80° C.

Observations for the Sika^®^ PS polyurethane:

- Relationship between the maximum tensile strength and elongation at break and the deformation speed; for the test temperature, the increase of the deformation speed resulted in the increase of the strength and limit deformation. The differences were particularly visible for measurements at 20 °C; at 80 °C, the relationship was kept, but the deformation increase was significantly lesser, and the difference between speed 10^−1^ mm/min and 10^1^ mm/min was only 3%;
- Relationship between the decrease of tensile strength and limit deformation as a result of elevated temperature; the temperature increase to 80 °C caused a reduction the mean stress regardless of the test speed, by 17% for v = 10^−1^ mm/min, 24% for v = 10^0^ mm/min and by 27% for v = 10^1^ mm/min, respectively;
- The least variability of stresses σ_M_ was obtained at 20 °C for the test speed 10^0^ mm/min (V = 2.0%), and at 80 °C for the test speed 10^1^ mm/min (V = 1.3%);
- The greatest variability of stresses σ_M_ and deformation ε_M_ was obtained at 20 °C for the test speed 10^1^ mm/min (V = 6.2% and V = 23.2%, respectively), and at 80 °C for the test speed 10^0^ mm/min (V = 8.2% and V = 7.8%, respectively).

**Figure 5:**
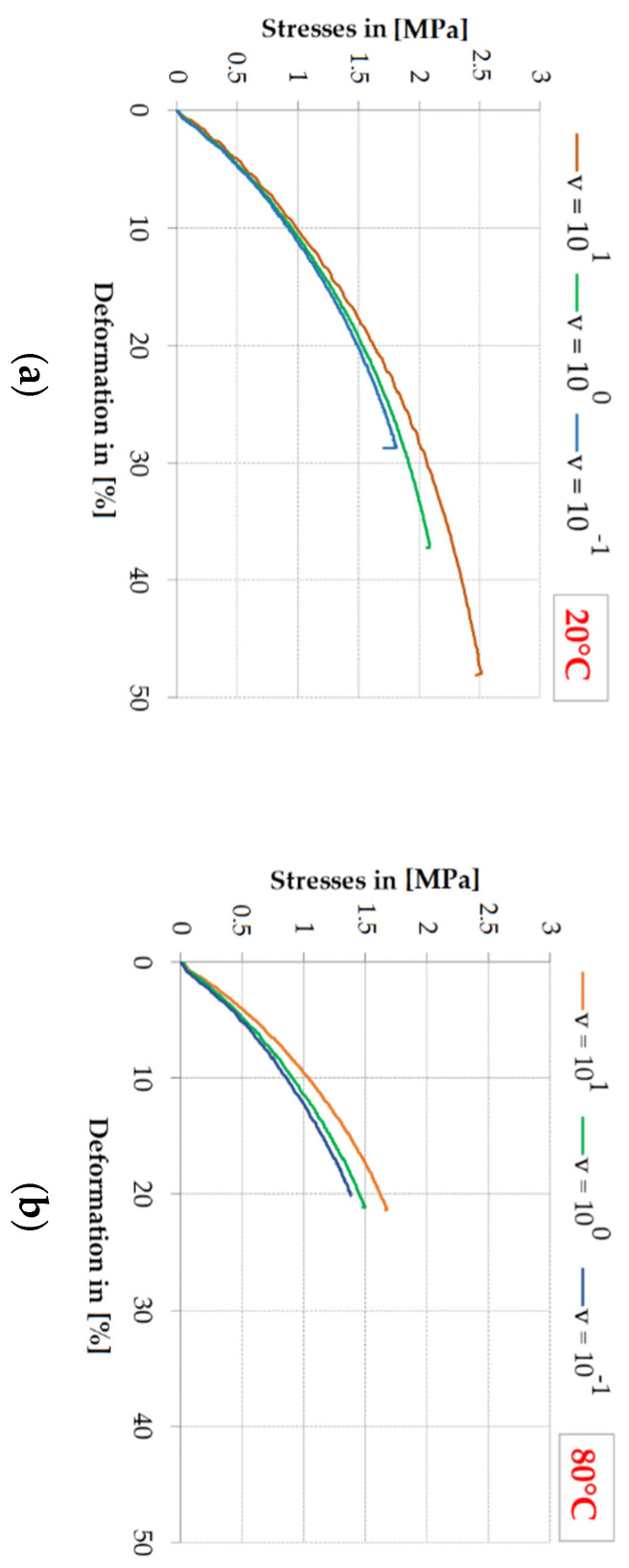
Impact of the test speed on the stress and limit deformation for selected representative specimens of PS adhesive: (**a**) at 20 °C; (**b**) at 80 °C.

Figure 5a,b shows the impact of stresses σ_M_ on deformation ε_M_ for selected tests (closest to the mean values) depending on the deformation speed, for two temperatures, for the Sika^®^ PS adhesive.

**Figure 6:**
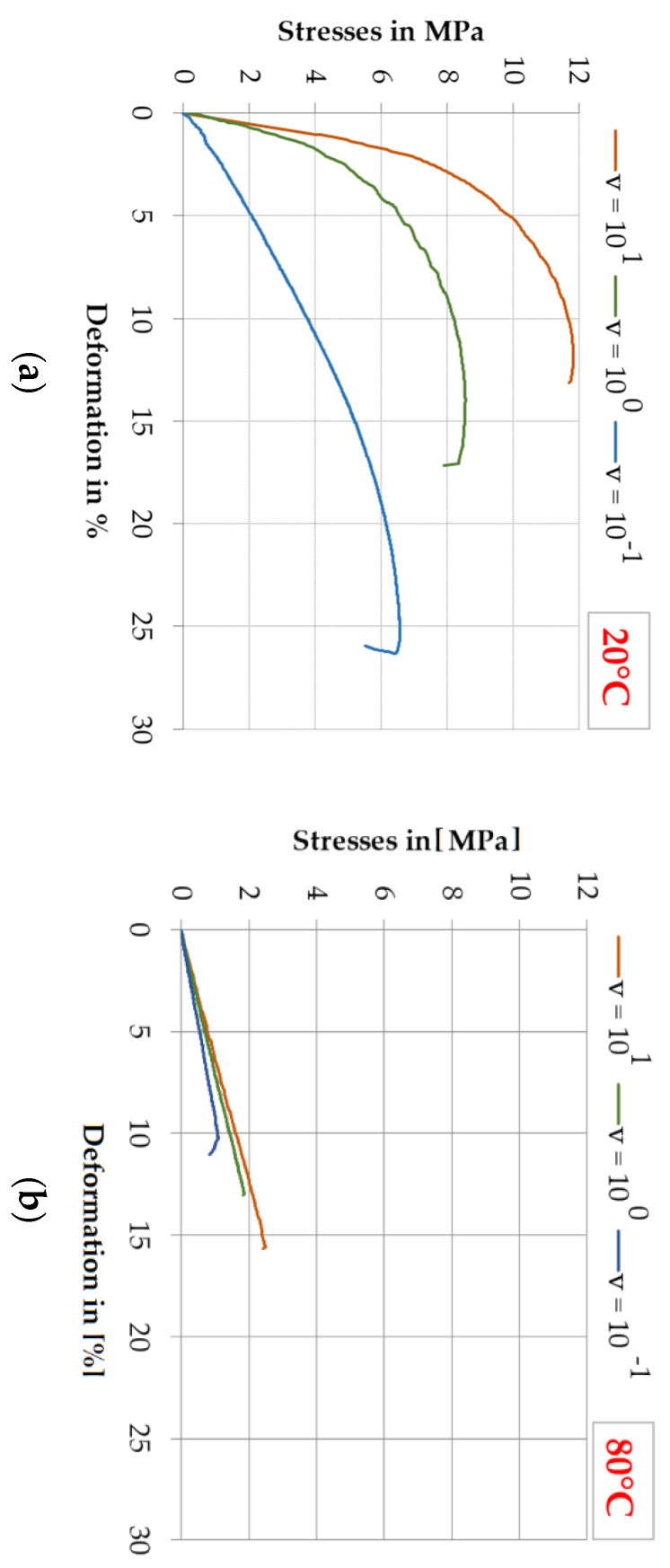
Impact of the test speed on the stress and limit deformation for selected representative specimens of EP 2579-1 adhesive: (**a**) at 20 °C; (**b**) at 80 °C.

Observations for the EP 2579-1 adhesive:

- At 20 °C, the test speed increase had an impact on the strength increase; however, contrary to Sika^®^ PS, the limit deformation decreased as shown in Figure 6a,b; quite high deformation values were obtained (taking into account the fact that this is an epoxy adhesive): above 10% for each speed. However, from the point of view of using the epoxy for joining wood components, greater deformability can be an advantage as it may allow for an increased ductility lacking in the wood characterized by brittle form of failure;
- At 80 °C, the epoxy adhesive showed the properties similar to that of the polyurethane, which is related to the glass transition taking place in the material (change of the elastic-brittle state to the flexible-rubbery state); the test speed increase caused the increase of the strength and limit deformability which also exceeded 10%;
- The least variability of stresses σ_M_ was obtained at 20 °C for the test speed 10^−1^ mm/min (V = 5.5%), and at 80 °C for the test speed 10^1^ mm/min (V = 13.4%);
- The greatest variability of stresses σ_M_ and deformation ε_M_ was obtained at 20 °C for the test speed 10^1^ mm/min (V = 7.7% and V = 13.2%, respectively) and at 80 °C for the test speed 10^−1^ mm/min (V = 29.7% and V = 13.8%, respectively).

Figure 6a,b shows the impact of stresses σ_M_ on deformation ε_M_ for selected tests (closest to the mean values) depending on the deformation speed, for two temperatures, for the epoxy adhesive.

At 20 °C for the EP 2579-1 adhesive, the stresses were much higher than for the Sika^®^ PS regardless of the deformation speed. For epoxy, the mean tensile stress values were higher than the mean values for polyurethane by: 252% for the speed v = 10^−1^ mm/min, 301% for the speed v = 10^0^ mm/min and by 381% for v = 10^1^ mm/min. However, at 80 °C the mean tensile stresses for the epoxy adhesive significantly drop, and their level is similar to that of the polyurethane. At v = 10^−1^ mm/min and 80 °C, the result for Sika^®^ PS (1.5 MPa) was greater than that of the epoxy by 66% (0.9 MPa). The obtained results indicate the lower variability of the Sika^®^ PS polyurethane adhesive at elevated temperature and the recorded decrease of stress values are not as large as for the epoxy adhesive.

The flexible adhesive Sika^®^ PS at the examined test temperatures was characterized by a very low variability of Young’s E-modulus (slope of the stress vs. deformation), especially for the deformation range from 0 to 10% which cannot be said about the samples made of epoxy EP 2579-1. Such a small variation in the E-modulus for Sika^®^ PS is due to the fact that the adhesive is in a rubbery state at 20 °C and 80 °C. The behaviour of the epoxide is influenced not only by the glass transition Tg, which causes a large drop in the stresses value, but also by the speed of the test. For higher speeds, less deformation is observed—greater stiffness, which is related to the behaviour of molecular chains at the micrometre level

The static compression test on cylindrical specimens with dimensions (l = h = 28 mm) included six measurements at each temperature for the deformation speed of v = 10^0^ mm/min. The results are given in Table 3 which includes the maximum compressive stresses values and maximum deformation (relative compression) values in statistical terms. The following were calculated for each temperature: mean compressive stress (m), standard deviation (s) and coefficient of variation (V).

At 20 °C, the values of mean compressive stresses for the EP 2579-1 were greater by 85% than those of the Sika^®^ PS. For 20 °C the PUR has the higher compression, at 80 °C it is the EP high the higher value.

**Figure 7:**
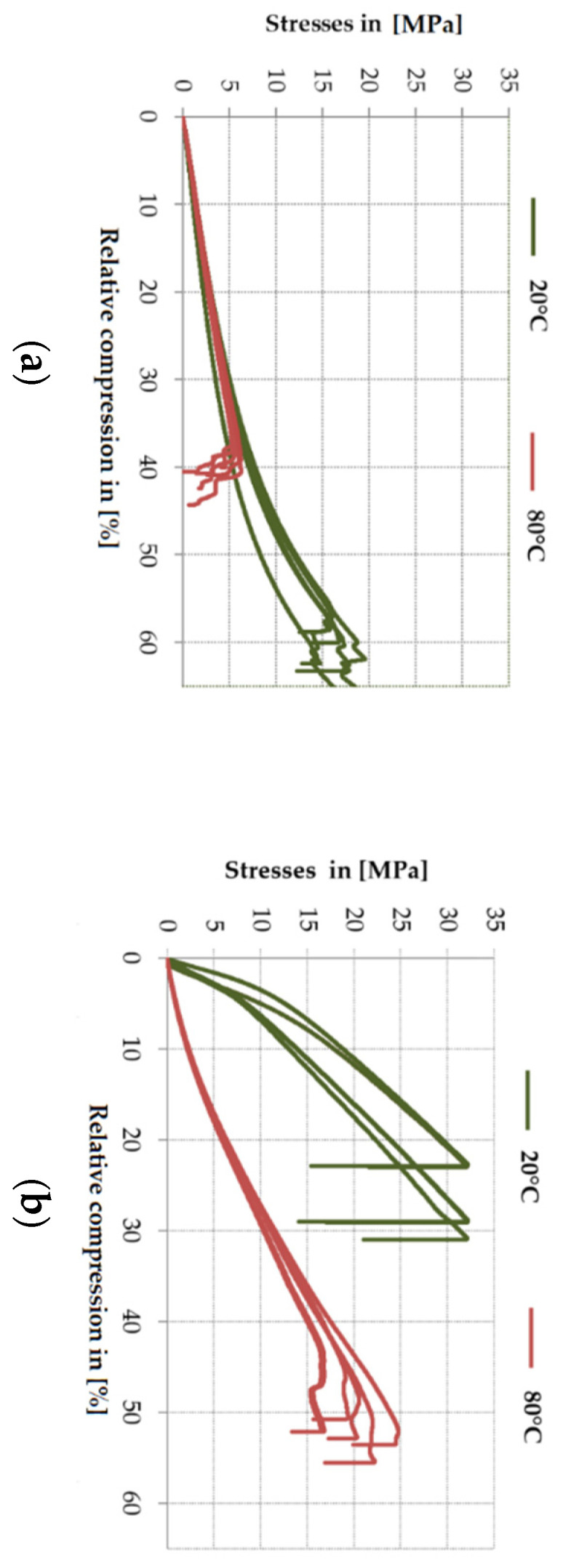
Impact of the test temperature on the stress and limit deformation in the compression test: (**a**) for PS; (**b**) for EP 2579-1.

For both adhesives, the mean compressive stress at 80 °C was reduced by 67% for the Sika^®^ PS and by 36% for the EP 2579-1 in comparison with the temperature of 20 °C. The diagrams below show the impact of stresses σ_M_ on relative compression ε_M_ for both adhesives and for both temperatures. As indicated by Figure 7a, in case of the Sika^®^ PS, the temperature increases to 80 °C resulted in the reduction of relative compression by 38% in relation to the measurement temperature of 20 °C. The situation for the epoxy EP 2579-1 was opposite which is shown in Figure 7b. At 80 °C, the mean relative shortening increased by 88%.

It was observed that for a rigid adhesive at 20 °C, the stresses curve vs. relative shortening after the initial proportional increase (linear-elastic range) “bends” and reaches the peak, determining the maximum stress, and then rapidly decreases. At a temperature of 80 °C above the Tg temperature, the epoxy begins to behave as a flexible adhesive, after reaching the maximum stress value, the material softens (the sample is barrel-shaped during the test—it has the shape of a barrel) and then disintegrates.

The hardness measurements results are presented in Table 4 as the mean values from six measurements, and the coefficients of variation (V in %) are given in parentheses. The measurements indicate that the Sika^®^ PS polyurethane has a stable hardness value at elevated temperature. The difference between 20 °C and 80 °C was 2 on the Shore A hardness scale. A significant decrease of hardness at 80 °C was observed for the EP 2579-1 epoxy resin. The decrease is related to the glass transition phenomenon (transition from the hard state to a rubbery state) which reduces the joint load capacity. When the specimens were cooled down to the ambient temperature (20 °C), the epoxy resin regained its original hardness (>100).

The impact of elevated temperature on the wood compressive strength parallel to the grain was also tested. The test of quasi-static compression parallel to the grain was performed on 20 mm × 20 mm × 30 mm specimens according to the standard [53]. The mean strength values calculated for 30 specimens, converted to the wood moisture content of 12% according to [53] are presented in Table 5.

As indicated by Table 5, the temperature increase to 80 °C affected the wood compressive strength parallel to the grain. The mean strength converted to 12% moisture content decreased by 27.72% in comparison with the strength at 20 °C. The obtained strength reduction corresponds to the results described in [54] and the recommendations included in the Eurocode 5 [55], according to which the strength reduction correction factor of k_temp_ = 0.8 should be used for wood structures in conditions of periodic occurrence of temperature higher than 75 °C.

### 3.2. Tests of Double Lap Shear Joints

The results of compression tests of double shear lap joints and the comparative analysis are given below. The impact of temperature and adhesive layer thickness of the joint load capacity was described. The shear joint failure type was compared. The failure types were described based on the PN-EN ISO 10365:1992 standard [56], using the following designations: cohesive failure in wood (ct), cohesive failure in polymer (cp), adhesive failure at the wood–polymer interface (at-p), and mixed failure (md). The destructive force value (F_max_) and the corresponding machine’s cross-beam displacement (dl/F_max_) were recorded during the tests and are presented in Table 6 along with the values of nominal shear stresses in the adhesive joint. In addition, Table 6 includes information on the failure form of the wood-polymer joint for each recorded trial.

In case of double shear lap joints made with the Sika^®^ PS adhesive at 20 °C the mean destructive force, depending on the adhesive layer thickness (t), was: 9.2 kN for t = 1 mm, 11.7 kN for t = 2 mm and 11.3 kN for t = 4 mm, respectively. g. Double shear lap joints of thickness t = 4 mm had the greatest destructive force and the least variability of results (V = 6.1%). It should be noted that the obtained results for the adhesive layer of thickness t = 4 mm were very similar, and the difference was only 4%. The greatest variability of F_max_ results was obtained for the joint of thickness t = 1 mm (V = 24.2%). Based on the results, it was concluded that the joint behaviour is stable in the thickness range from 2 to 4 mm.

**Figure 8:**
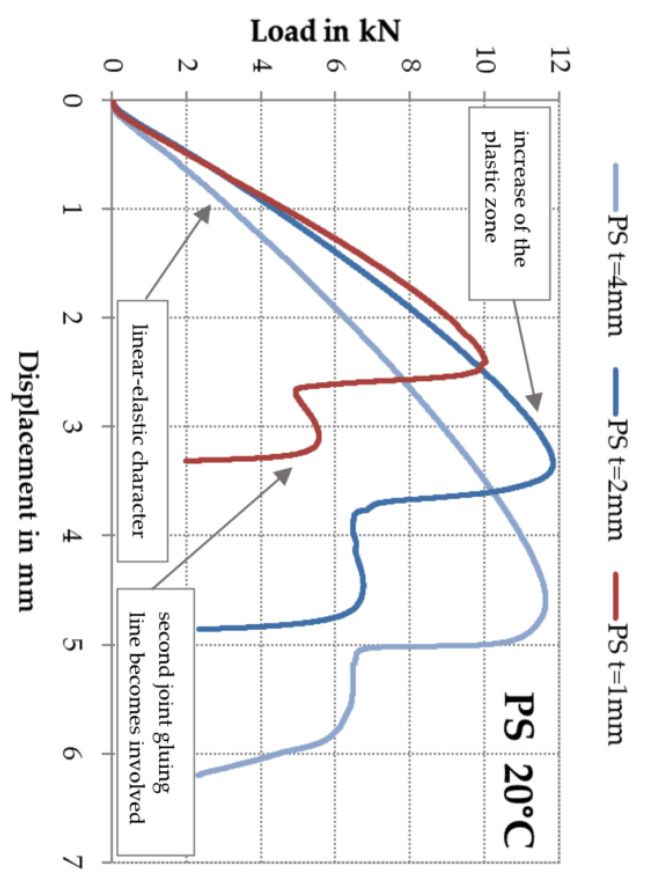
Force vs. displacement plot for various adhesive layer thicknesses t = 1, 2 and 4 mm at 20 °C.

In addition, the following relationship may be indicated: when the adhesive layer thickness increases, the corresponding vertical displacement (dl/F_max_) recorded for the destructive force also increases. The greatest mean value equal to dl/F_max_ = 4.5 mm was recorded for the adhesive layer of thickness t = 4 mm at standard deviation s = 0.2 mm. For the t = 1 mm thickness, the mean vertical displacement was 2.2 mm at standard deviation s = 0.3 mm. Despite high variability of results in terms of destructive force, as described above, the variability of displacement values was low (V = 12%). Figure 8 presents the relationship between the adhesive layer thickness and the displacement at 20 °C. The plots show the plastic zone occurring before the failure and the involvement of the second adhesive bonding line after the first one was damaged. In addition, it was observed that the failure energy grew with the increasing adhesive layer thickness. The difference in the slope of the load vs. the displacement between the individual thicknesses of the adhesive layer (except that for t = 4 mm the bond line is more elastic, which causes the higher deformation) is caused by a change in the shear account in the connection.

**Figure 9:**
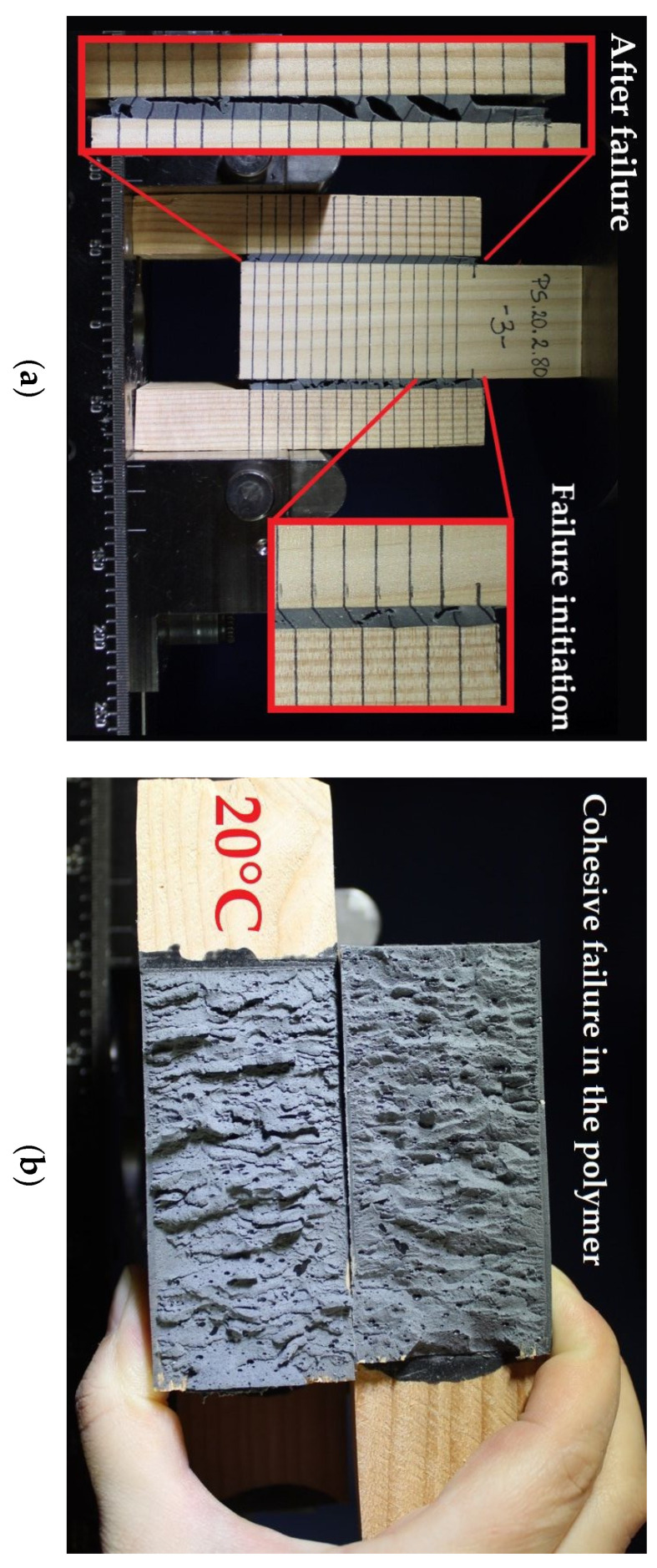
Reference specimen: (**a**) failure initiation and the joint after failure; (**b**) joint cross section—cohesive failure in polymer.

The failure of the Sika^®^ PS joint occurred regardless of its thickness t = 1, 2 or 4 mm (cohesive failure in polymer (cp)). A representative example of failure initiation and the joint failure after failure for thickness t = 2 mm at the test temperature of 20 °C is shown in Figure 9. In the vast majority of double shear lap joints, the failure initiated at the joint ends (stresses concentration areas) and proceeded towards the joint centre.

In the double shear lap joints with the EP-2579-1 adhesive for the test temperature of 20 °C, the mean destructive force depending on the adhesive layer thickness (t) was equal to 35.2 kN for t = 1 mm, 22.1 kN for t = 2 mm and 42.8 kN for t = 4 mm, respectively. In comparison with the Sika^®^ PS joint, the destructive force increased by 282% for t = 1 mm, 88% for t = 2 mm and by 279% for t = 4 mm. Table 6 indicates that the greatest value of the destructive force was recorded for the series of six specimens with adhesive layer thickness t = 4 mm for which the standard deviation was s = 2.6 kN.

**Figure 10:**
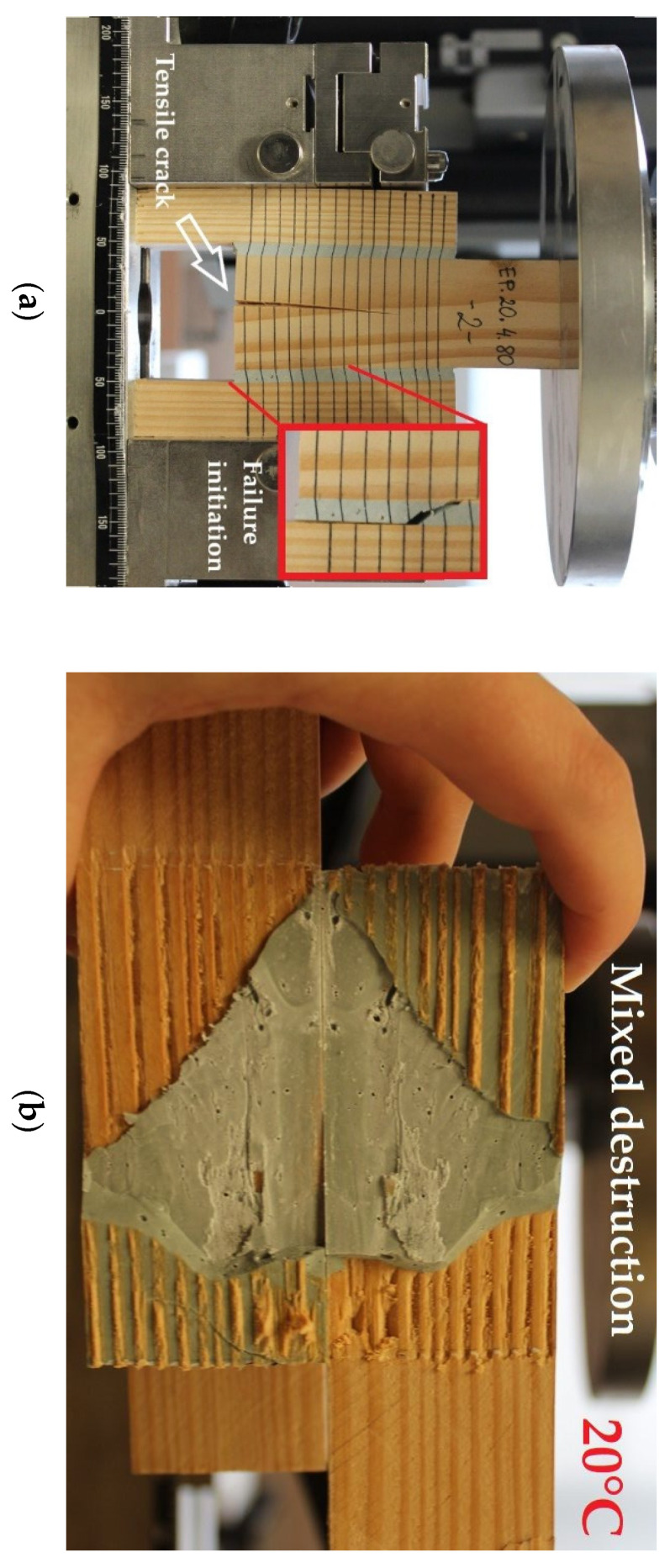
Failure of the reference t = 4 mm specimen: (**a**) premature failure as a result of tension perpendicular to the grain and failure initiation; (**b**) join cross section—mixed failure (adhesive failure on the wood-polymer interface and cohesive failure in the polymer).

It should be noted that due to theoretically rigid character of the epoxy adhesive the greatest values of destructive forces were expected for the adhesive layer thickness of t = 1 mm and not for t = 4 mm. The authors are cautious about the obtained results because the dominating factor affecting the joint failure, particularly for the thickness t = 2 mm, was the strength of the wood itself. The obtained results may have been affected by the heterogeneity of the wood. During the tests of the t = 4 mm adhesive layer thickness, a premature failure of the bottom end of the central component was observed as a result of the occurrence of horizontal tensile stresses, perpendicular to the grain, which is shown in Figure 10a.

**Figure 11:**
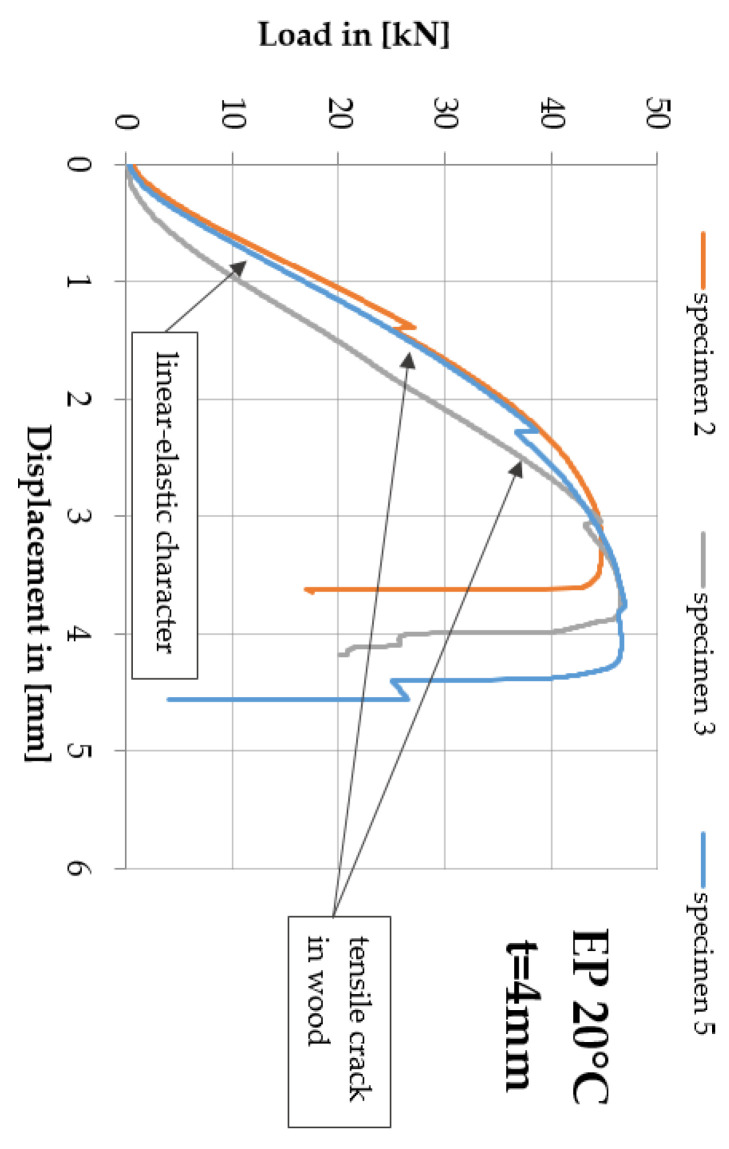
Force vs. displacement plot for selected specimens EP2579-1 double shear lap joint for the adhesive layer thickness t = 4 mm at 20 °C.

These cracks did not affect the final behaviour of the double shear lap joint, nevertheless irregularity was observed on some force vs. displacement plots which is shown in Figure 11. In addition, it was noticed that for the t = 1 mm adhesive layer the failure occurred in the wood or was of the mixed type.

It has been observed the downward for the rigid and flexible adhesives trends are different from each other. For the double shear lap joint with the rigid adhesive, after reaching the peak, the applied loading F_max_ drops to zero sharply with less plastic deformation. On the other hand, in the case of the double lap joint adopting the flexible adhesive, the loading F_max_ decreases quite slowly experiencing long displacement until to final failure.

Analogous tests for the double shear lap joint were performed at 80 °C and their results are presented in Table 7 which includes information on the failure form of the wood-polymer joint for each recorded trial.

**Figure 12:**
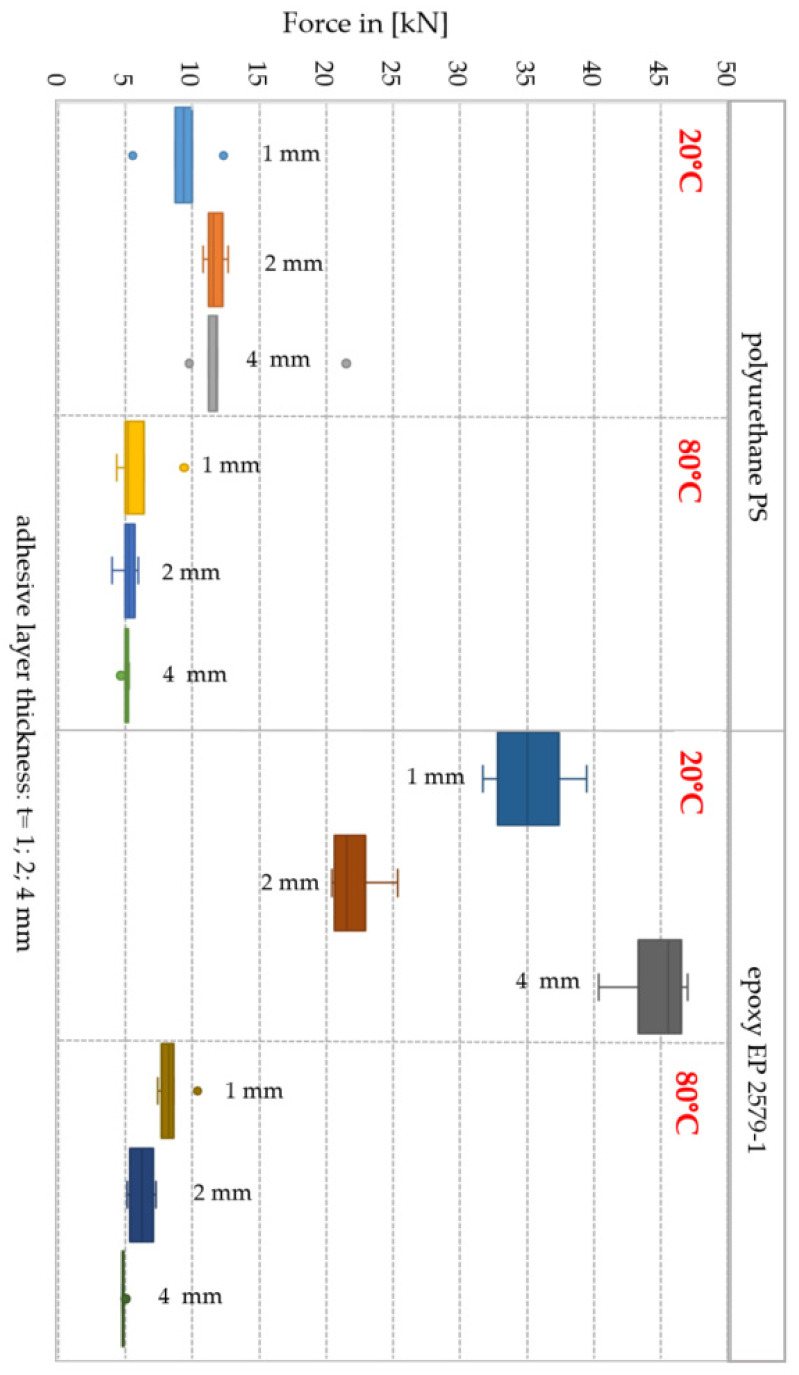
Box and whisker plots for the maximum destructive force at 20 °C and 80 °C.

Box and whisker plots (summarizing all the results) for the trials at 20 °C and 80 °C are presented below in Figure 12.

In case of both adhesives at the test temperature of 80 °C, the mean destructive force was lower than at 20 °C. For the Sika^®^ PS double lap joint of the thickness t = 1 mm, the mean destructive force was 5.3 kN at standard deviation of s = 0.7 kN, which constituted 57% of the mean force F_max_ for 20 °C. The greatest decrease of the mean destructive force, and consequently the greatest load capacity decrease, was observed for the t = 2 mm joint (by 55%). At 80 °C, the increase of the thickness of the adhesive layer resulted in a decrease in the load-bearing capacity of the connection. The destructive force for Sika^®^ PS and thickness adhesive layer t = 4 mm was 5.1 kN. The least variability of the shear stresses in the joint was for the t = 1 mm joints (V = 4.1%). For the remaining thicknesses, the coefficient of variation (V) was close to 13%

**Figure 13:**
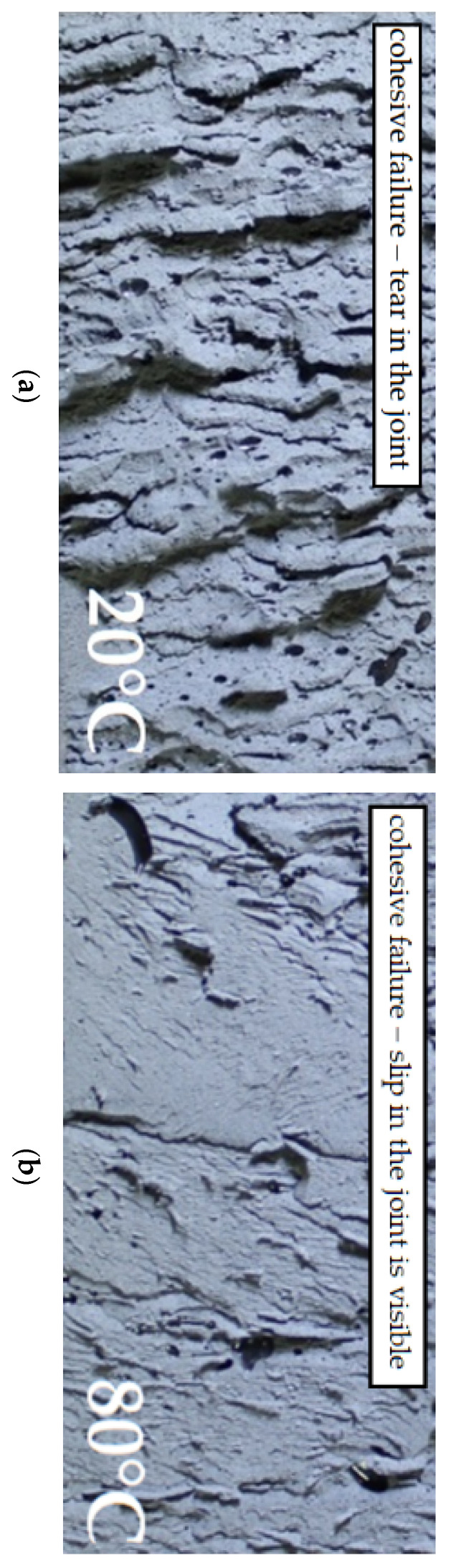
Failure type in the SikaPS epoxy adhesive joint: (**a**) at 20 °C; (**b**) at 80 °C.

Similar to the testing of only adhesives, the tests of double lap joints indicate a clear impact of temperature on deformability. The mean value of displacement read from the machine’s cross beam for Sika^®^ PS decreased by 67% for t = 1 mm, 51% for t = 2 mm and by 52% for t = 4 mm. The failure type at 80 °C was the same as at 20 °C, i.e., it was the cohesive failure in polymer (cp). The difference was the structural changes in the polymer on the macro scale as a result of temperature which is shown in Figure 13. As shown in Figure 13, the temperature increase causes the joint plasticization—a smoother failure form (absence of distinctly torn material surface which occurred at 20 °C), a slip in the joint is visible in the cross section.

**Figure 14:**
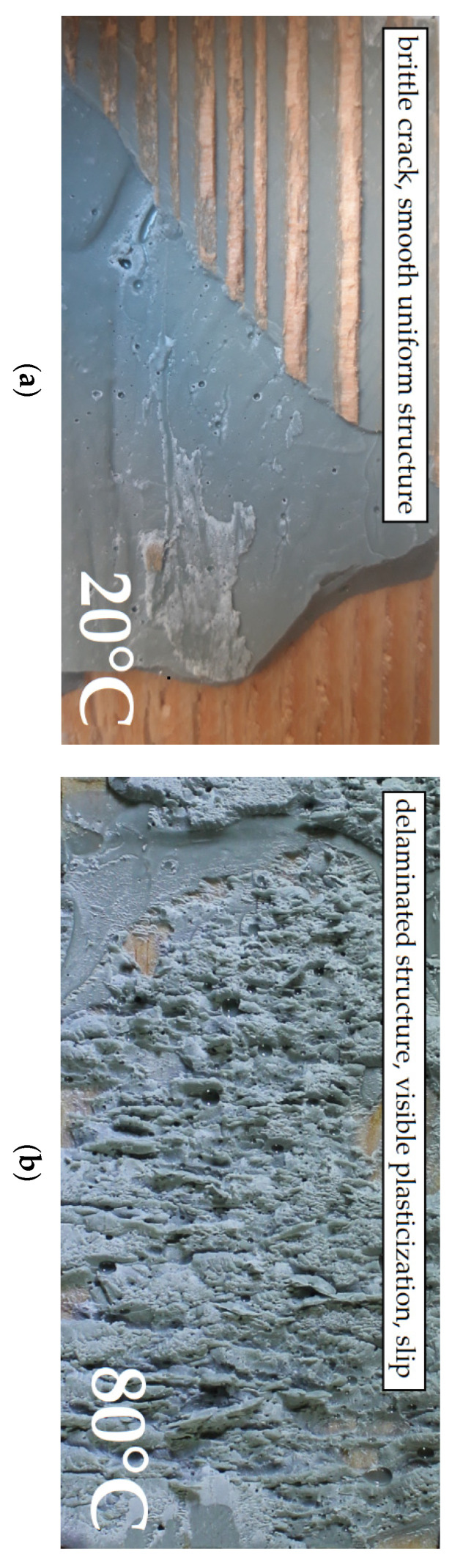
Failure form in the EP 2579-1 epoxy joint: (**a**) at 20 °C; (**b**) at 80 °C.

The double lap joints made with the EP 2579-1 epoxy experienced the destructive force decrease in relation to the temperature of 20 °C by 76% for t = 1 mm, 72% for t = 2 mm and by 89%, respectively. In the t = 4 mm double lap joint, at 80 °C the load capacity was reduced by almost 11% in comparison to the load capacity at 20 °C. The analysis of the impact of joint thickness on the load capacity at 80 °C indicates the load capacity decrease when the joint thickness increased in relation to the results for t = 1 mm (F_max_ = 8.4 kN): by 26% for t = 2 mm, and by 42% for t = 4 mm, respectively. The failure form at 80 °C was different. A cohesive failure in epoxy (cp) was observed. The differences in the adhesive joint structure are presented in Figure 14. As seen in Figure 14, at 80 °C the failure changes from brittle to plastic. The failure occurred as a result of tear in the material with a visible slip.

**Figure 15:**
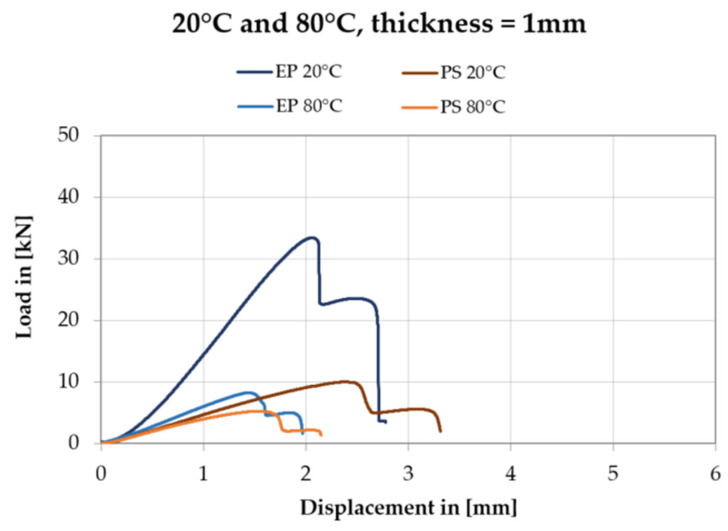
Force vs. displacement plots for Sika^®^ PS and EP 2579-1 adhesives with the thickness t = 1 mm, at temperature 20 °C and 80 °C.

**Figure 16:**
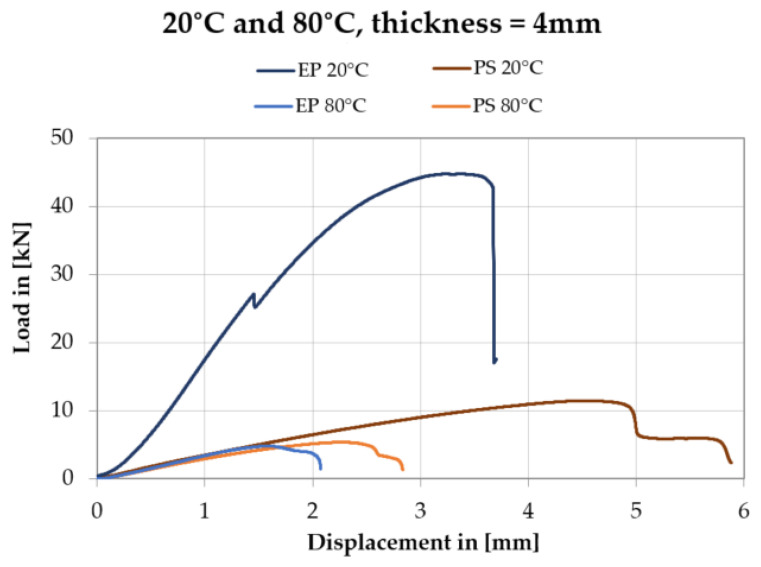
Force vs. displacement plots for Sika^®^ PS and EP 2579-1 adhesives with the thickness t = 4 mm, at temperature 20 °C and 80 °C.

The results of shear tests of the double shear lap joint for the Sika^®^ PS and the EP 2579-1 are presented as force vs. displacement plots in the same scale for the chosen joint thicknesses (t = 1 mm and t = 4 mm) at two temperature values. Figure 15 and Figure 16 show only one representative curve (close to the mean value) for the four cases (at 20 °C and 80 °C).

## 4. Conclusions

The paper presents the results of experimental tests conducted at 20 and 80 °C for a double lap joint made with the use of two adhesive types, the polyurethane and epoxy in three joint thicknesses. The destructive compression tests were conducted under a quasi-static load. In addition, the paper includes the results of strength tests of materials comprising the joint. The following conclusions can be drawn from the test results presented in the paper:

- It was proven that for the Sika^®^ PS flexible adhesive, both at 20 °C and 80 °C, the increase of deformation speed results in the increase of strength and decrease of deformability; however, for the epoxy adhesive, this relationship is true only for the test temperature of 20 °C;
- The type and thickness of adhesive used in wood structures has a significant impact on the load capacity and behaviour of the adhesive joint at elevated temperature;
- The rigid joint (with epoxy adhesive) in comparison to the flexible joints (with PUR adhesive) at 20 °C, depending on the joint thickness (t), has greater destructive force values by 282% for t = 1 mm, 88% for t = 2 mm, and 279% for t = 4 mm, respectively;
- As the temperature increases, the load capacity of the shear double lap joint decreases, and the percent of decrease is greater for the epoxy adhesive than for the flexible adhesive in comparison with 20 °C;
- The temperature increase affects the form of epoxy failure (change from cohesive failure in the wood or mixed failure to the cohesive failure in the joint), but the failure form in the polyurethane adhesive joint is invariable and stable;
- At 80 °C, the greatest value of destructive force (F_max_ = 5.3 kN) in double lap joints made with the Sika^®^ PS flexible adhesive was obtained for the layer thickness t = 1 mm and t = 2 mm; for the EP 2579-1 epoxy adhesive and the layer thickness t = 4 mm, the destructive force was F_max_ = 4.9 kN which is 84% of the flexible joint load capacity. It can be said that for an appropriate joint thickness at 80 °C the load capacity of a flexible joint is higher and consequently the flexible does not have such a large decrease;
- In a flexible double lap joint, the temperature increase results in a decrease of mean displacement values by about 50–69%, depending on the joint thickness;
- In the case of joining wood with thick adhesive layers, where high deformation is desired, the damage will take place in the adhesive layer, thus the joint’s load-bearing capacity will be designed taking into account the strength of the bonding line, not the wood.

The further research will focus on the methods of securing the wood structures with the use of bonding technology by reducing the concentration of stresses which occur in traditional dowel-type joints and the improvement of the structural plasticity in the context of temperature increase. The decrease of mechanical parameters in adhesive joints as a result of elevated temperature has a key importance in the design process (ensuring safety during the long use for many years.

## 5. Patents

Patent nr PL207028 (B1): “Method of making the load-bearing repair joints of set mechanical parameters in concrete and masonry building structures”, protected by the Owner as the patent No. PL207028 (B1) granted by the Patent Office of the Republic of Poland and announced on 29.10.2010 WUP 10/10, as a result of patent application P-370025 submitted to the Patent Office of the Republic of Poland on 10.09.2004 and implemented on the basis of the granting of the full and exclusive licence to FlexAndRobust Sp. z o.o. by the Cracow University of Technology.

## Figures and Tables

**Table 1:** Parameters of adhesives.

Parameter	Sika^®^ PS	EP 2579-1
density	1.4 g/cm^3^	1.6 g/cm^3^
viscosity	Component A 4.00 Pa·s	Component A 500,000 mPa·s
	Component B 0.26 Pa·s	Component B 140,000 mPa·s
shear strength 14 days	2.8 MPa	13.3 MPa
operating temperature	−40 °C to +80 °C	−55 °C to +100 °C
pot life	15 min. at 20 °C	60 min. at 23 °C

**Table 2:** The results for uniaxial tensile test at 20 °C and 80 °C.

Adhesive Type and Temperature		Stresses σ_M_ in MPa			Deformation ε_M_ in %		
Test Speed (mm/min)		10^−1^	10^0^	10^1^	10^−1^	10^0^	10^1^
PS 20 °C	m	1.8	2.1	2.5	29.2	37.5	43.4
	s	0.1	0.0	0.2	2.5	5.0	10.1
	V in %	5.8	2.0	6.2	8.6	13.3	23.2
PS 80 °C	m	1.5	1.6	1.8	20.5	20.9	23.4
	s	0.1	0.1	0.0	0.5	1.6	1.0
	V in %	4.3	8.2	1.3	2.6	7.8	4.3
EP-2579-1 20 °C	m	6.4	8.3	11.8	25.9	17.3	12.4
	s	0.4	0.6	0.9	1.4	1.4	1.6
	V in %	5.5	6.7	7.7	5.6	8.3	13.2
EP-2579-1 80 °C	m	0.9	1.9	2.2	8.7	13.0	13.9
	s	0.23	0.3	0.3	1.2	1.4	1.8
	V in %	29.7	14.6	13.4	13.8	10.7	12.6

**Table 3:** Results for uniaxial compression test at 20 °C and 80 °C.

Adhesive Type and Temperature		Stresses ^1^ σ_M_ in MPa	Relative Compression ^1^ ε_M_ in %
PS 20 °C	m	17.4	62.3
	s	1.6	4.7
	V in %	9.2	7.6
PS 80 °C	m	5.7	38.4
	s	0.4	1.2
	V in %	7.5	3.3
EP-2579-1 20 °C	m	32.2	26.9
	s	0.0	3.8
	V in %	0.1	14.1
EP-2579-1 80 °C	m	20.7	50.5
	s	3.1	3.6
	V in %	14.9	7.1

^1^ Test speed 10^0^ mm/min.

**Table 4:** Mean values of Shore A scale hardness for 20 °C and 80 °C.

Material	Shore A Scale Hardness	
Temperature	20 °C	80 °C
PS	89 (5.1%)	87 (6.3%)
EP 2579-1	>100	85 (6.1%)

**Table 5:** Results of wood compressive strength tests parallel to the grain at 20 °C and 80 °C.

Temperature		20 °C	80 °C
Compressive strength parallel to the grain R_c,0_^1^	m	56.60	40.91
	s	5.00	8.19
	V in %	8.83	20.02

^1^ Converted to the wood moisture content of 12%.

**Table 6:** Shear test results for the double lap joint during compression at 20 °C.

Test Temperature 20 °C										
Specimen Type (Adhesive Layer Thickness)		Specimen No.						m ^1^	s ^2^	V (%) ^3^
		1	2	3	4	5	6			
PS (t = 1 mm)	F_max_ (kN)	8.8	10.0	8.7	12.3	5.5	9.9	9.2	2.2	24.2
		cp	cp	cp	cp	cp	cp			
	dl/F_max_ (mm)	2.3	2.4	2.3	2.6	1.9	1.9	2.2	0.3	12.1
	Shear strength (MPa)	1.4	1.6	1.4	1.9	0.9	1.6	1.4	0.4	24.4
PS (t = 2 mm)	F_max_ (kN)	11.1	12.7	10.8	11.8	12.4	11.7	11.7	0.7	6.1
		cp	cp	cp	cp	cp	cp			
	dl/F_max_ (mm)	3.1	3.5	3.4	3.5	3.6	3.0	3.4	0.2	6.9
	Shear strength (MPa)	1.7	2.0	1.7	1.9	1.3	1.8	1.8	0.1	6.2
PS (t = 4 mm)	F_max_ (kN)	11.7	11.1	11.8	11.9	11. 5	9.8	11.3	0.8	7.0
		cp	cp	cp	cp	cp	cp			
	dl/F_max_ (mm)	4.5	4.7	4.6	4.5	4.6	4.2	4.5	0.2	3.3
	Shear (MPa)	1.8	1.7	1.8	17	1.8	1.5	18	0.1	6.9
EP 2579-1 (t = 1 mm)	F_max_ (kN)	31.7	33.4	36.7	37.6	39.4	32.6	35.2	3.1	8.8
		ct	ct	md	md	md	ct			
	dl/F_max_ (mm)	2.2	2.1	2.3	2.4	2.3	2.5	2.3	0.2	6.8
	Shear strength (MPa)	4.9	5.2	5.7	5.9	6.2	5.1	5.5	0.5	8.9
EP 2579-1 (t = 2 mm)	F_max_ (kN)	25.3	20.4	22.4	20.6	23.1	20.6	22.1	1.9	8.7
		ct	ct	ct	md	ct	ct			
	dl/F_max_ (mm)	3.6	2.0	2.7	2.2	3.5	2.2	2.7	0.7	26.0
	Shear strength (MPa)	4.0	3.2	3.5	3.2	3.6	3.2	3.5	0.3	8.8
EP 2579-1 (t = 4 mm)	F_max_ (kN)	42.8	44.8	46.6	46.4	47.0	40.4	44.7	2.6	5.8
		md	md	at-p	at-p	md	at-p			
	dl/F_max_ (mm)	2.8	3.2	2.9	4.2	3.8	2.8	3.3	0.6	17.6
	Shear strength (MPa)	7.0	7.0	7.3	7.2	7.4	6.3	7.0	0.4	5.8

m ^1^—mean, s ^2^—standard deviation, V(%) ^3^—coefficient of variation.

**Table 7:** Shear test results for the double lap joint during compression at 80 °C.

**Test Temperature 80 °C**										
Specimen Type (Joint Thickness)		Specimen No.						m ^1^	s ^2^	V (%) ^3^
		1	2	3	4	5	6			
PS (t = 1 mm)	F_max_ (kN)	6.4	5.3	5.2	4.4	5.25	4.95	5.25	0.65	12.34
		cp	cp	cp	cp	cp	cp			
	dl/F_max_ (mm)	1.64	1.71	1.38	1.57	1.53	1.51	1.56	0.11	7.31
	Shear strength (MPa)	0.99	0.83	0.81	0.69	0.82	0.77	0.82	0.10	12.03
PS (t = 2 mm)	F_max_ (kN)	5.68	5.09	4.93	5.73	4.05	5.99	5.25	0.71	13.56
		cp	cp	cp	cp	cp	cp			
	dl/F_max_ (mm)	1.93	1.42	1.75	1.74	1.72	1.67	1.71	0.17	9.69
	Shear strength (MPa)	0.89	0.80	0.77	0.89	0.63	0.94	0.82	0.11	13.71
PS (t = 4 mm)	F_max_ (kN)	5.0	5.1	5.3	4.7	5.2	5.1	5.07	0.2	4.1
		cp	cp	cp	cp	cp	cp			
	dl/F_max_ (mm)	2.1	2.3	2.4	1.9	2.1	2.2	2.2	0.2	8.1
	Shear (MPa)	1.0	1.1	1.1	1.0	1.1	1.1	1	0.1	4.9
EP 2579-1 (t = 1 mm)	F_max_ (kN)	10.41	8.73	7.56	8.2	8.27	7.42	8.43	1.08	12.85
		cp	cp	cp	cp	cp	cp			
	dl/F_max_ (mm)	1.68	1.81	1.31	1.46	1.32	1.71	1.55	0.21	13.81
	Shear strength (MPa)	1.63	1.36	1.18	1.28	1.29	1.16	1.32	0.17	12.95
EP 2579-1 (t = 2 mm)	F_max_ (kN)	7.13	7.00	7.26	5.53	5.27	5.12	6.22	1.01	16.25
		cp	cp	cp	cp	cp	cp			
	dl/F_max_ (mm)	1.54	1.60	1.87	1.65	2.16	1.73	1.76	0.23	12.95
	Shear strength (MPa)	1.11	1.09	1.13	0.86	0.82	0.80	0.97	0.16	16.20
EP 2579-1 (t = 4 mm)	F_max_ (kN)	4.88	4.77	4.78	5.04	4.87	2.34	4.87	0.11	2.23
		md	md	cp	cp	cp	-			
	dl/F_max_ (mm)	1.99	1.09	1.81	1.58	1.04	1.30	1.50	0.42	28.29
	Shear strength (MPa)	0.76	0.74	0.75	0.79	0.76	0.37	0.76	0.02	2.46

m ^1^—mean, s ^2^—standard deviation, V (%) ^3^—coefficient of variation.

## Data Availability

The data presented in this study are available on request from the corresponding author. The data are not publicly available due to the ongoing work on the doctoral dissertation.
